# Intestinal bacteria—a powerful weapon for fungal infections treatment

**DOI:** 10.3389/fcimb.2023.1187831

**Published:** 2023-06-02

**Authors:** Liu Cong, Chaoqun Chen, Shanshan Mao, Zibing Han, Zuobin Zhu, Ying Li

**Affiliations:** ^1^ School of Medical Technology, Xuzhou Medical University, Xuzhou, Jiangsu, China; ^2^ Department of Genetics, Xuzhou Medical University, Xuzhou, Jiangsu, China

**Keywords:** fungal resistance, probiotics, antifungal, toxicity factor, active metabolites

## Abstract

The morbidity and mortality of invasive fungal infections are rising gradually. In recent years, fungi have quietly evolved stronger defense capabilities and increased resistance to antibiotics, posing huge challenges to maintaining physical health. Therefore, developing new drugs and strategies to combat these invasive fungi is crucial. There are a large number of microorganisms in the intestinal tract of mammals, collectively referred to as intestinal microbiota. At the same time, these native microorganisms co-evolve with their hosts in symbiotic relationship. Recent researches have shown that some probiotics and intestinal symbiotic bacteria can inhibit the invasion and colonization of fungi. In this paper, we review the mechanism of some intestinal bacteria affecting the growth and invasion of fungi by targeting the virulence factors, quorum sensing system, secreting active metabolites or regulating the host anti-fungal immune response, so as to provide new strategies for resisting invasive fungal infection.

## 1 Introduction

Invasive fungal infections (IFI) are an increasingly serious public health problem, which can cause prominent invasive lesions with serious symptoms in the host, such as life-threatening bloodstream infection and organ dysfunction (Bongomin et al., 2017). The most common clinical pathogenic fungi are *Candida albicans*, *Aspergillus fumigatus* and *Cryptococcus neoformans*. With the increasing number of immunosuppressed people caused by organ transplantation, cancer treatment, HIV and so on, the morbidity and mortality of IFI are rising gradually (Kato et al., 2018; Mayer and Kronstad, 2019). IFI are easy to relapse and difficult to cure, killing more than 1.5 million people worldwide every year, almost as many as tuberculosis (Bongomin et al., 2017). Moreover, IFI can lead to intestinal fungal dysbiosis, which in turn has a certain effect on metabolic diseases and intestinal disorders diseases such as gastric cancer, inflammatory bowel disease (IBD), irritable bowel syndrome (IBS), celiac disease (CeD), colorectal cancers etc. (**Table 1**) (Ramirez-Garcia et al., 2016; Li et al., 2019).

**Table 1:** Detrimental roles of fungi in the human gastrointestinal tract on metabolic diseases and intestinal disorders diseases.

Diseases	Related fungi	Related mechanism	Reference
Inflammatory bowel disease(IBD)	*Candida albicans* ↑ *Candida tropicalis* ↑ *Saccharomyces cerevisiae* ↓	Production of anti-glycan antibody such as anti-*S. cerevisiae* antibodies (ASCA) can be induce during colonization or infection of *C. albicans*	(Sendid et al., 2009)
Irritable bowel syndrome (IBS)	*Candida albicans* ↑ *Saccharomyces cerevisiae* ↑	After fungal stimulation, Dectin-1/Sy pathway drives visceral allergic reaction and mast cells release more histamine.	(Pimentel and Lembo, 2020)
Colorectal cancers	*Candida albicans* ↑ *Candida tropicalis* ↑	*C. albicans* mediates the up-regulation of macrophage glycolytic pathway, and up-regulates the expression and secretion of IL-7, and then induces type 3 intrinsic lymphocytes (ILC3) to express high levels of IL-22 through AhR and STAT3 pathways, which aggravates the progression of colorectal cancer.	(Wong and Yu, 2019)
Celiac disease(CeD)	*Candida albicans* ↑	*C. albicans* virulence factor, hyphal wall protein 1 (HWP1), is the same as or highly homologous to celiac-related-α and γ gliadin in t-cell epitope and acts as a substrate for transglutaminase (TG), assisting in the production of autoreactive antibodies	(Chibbar and Dieleman, 2019)

“↑”indicates an increase in abundance; ”↓”indicates a decrease in abundance.

However, the current antifungal drugs are limited and outdated. Only three types of antifungal drugs are the first line drugs to treat human systemic fungal infections (**Table 2**). Amphotericin B, the first discovered polyene anti-fungal agent, binds to the ergosterol of cell membrane and “strips” it out, thereby destroying the structure of the fungal cell membrane. While the high toxicity limits its clinical application. Later, azoles, novel anti-fungal agents with lower side-effect, were developed, that can block the ergosterol synthesis by inhibiting lanosterol 14-α-demethylase (encoded by *ERG11*) (Perfect, 2017). However, due to the agricultural use of azoles to protect crops from fungal infection, azoles resistance gradually developed in the 1990s (Verweij et al., 2020). The third-generation antifungal drug echinocandins (such as caspofungin) work by blocking the biosynthesis of β-(1,3) d-glucan (encoded by *FKS1*), an essential component of fungal cell walls. Unfortunately, echinocandin-resistant *Candida* species have emerged in both laboratory and clinical settings (Medici and Del Poeta, 2015).

**Table 2:** First-line antifungal drugs and their limitations.

First-line antifungal drugs	Action target	Limitations	Reference
Amphotericin B	Fungal ergosterol	Side effects such as fever, heart inflammation and kidney problems are even life-threatening	(Sabra and Branch, 1990)
Azole drugs	Lanosterol 14-α- demethylase	Inhibit cytochrome P450 (a drug metabolizing liver enzyme needed for detoxification of human blood); Liver toxicity caused by combination with other drugs	(Marx-Stoelting et al., 2020)
Echinocandins	β-(1,3) D-glucan	Be administered once a day, and each infusion needs to last for one hour; Low bioavailability; Ocular toxicity	(Patil and Majumdar, 2017)

Furthermore, the widespread use of clinically antifungal agents has accelerated fungal resistance rates (Fairlamb et al., 2016). *Candida auris* is a superbug that first reported in Japan in 2009 (Wang et al., 2020). Although the exact mode of transmission is unclear, *C. auris* can rapidly affect patients and colonize the skin persistently (Schelenz et al., 2016; Lockhart et al., 2017; Vallabhaneni et al., 2017). Like other fungi, it can cause superficial and invasive candidiasis as well as bloodstream infections (Mane et al., 2016; Chowdhary et al., 2017). In addition, *C. auris* has multi-drug resistance to two or more classes of drugs and a few (about 4%) to all classes of antifungal drugs (Sanguinetti et al., 2015). Echinocandins are currently commonly used to treat *C. auris* infections, but caspofungin has been proved to be ineffective against *C. auris* biofilms (Harriott et al., 2010; Pahwa et al., 2014).

Meanwhile, COVID-19, caused by severe acute respiratory syndrome coronavirus 2 (SARS-CoV-2), emerged at the end of 2019 and since then has spread worldwide (Gorbalenya et al., 2020; Zhou et al., 2020). When the patient’s condition worsens, hospitalization is required and eventually intubation may be required and the patient is transferred to the intensive care unit (Zhu et al., 2020). The respiratory failure associated with novel coronavirus infection is the main driver of death in this population; However, some observations indicate that patients with COVID-19 may also have a higher risk of secondary infections (Rawson et al., 2020). For example, patients can further develop fungal infections (eg, IFI) at an advanced stage of the disease, especially in severely ill or immunocompromised patients (Guo et al., 2019). In China, Chen et al. performed fungal culture on admission to all 99 patients and identified 5 cases (5%, 5/99) of fungal infection, including 1 case of *Aspergillus flavus*, 1 case of *Candida glabrata* and 3 cases of *C. albicans* (Chen et al., 2020). Yang et al. found that the presence of *A. flavus*, *A. fumigatus* and *C. albicans* in all 52 critical patients (3/52, 5.8%) (Yang et al., 2020). Clinical abuse of broad-spectrum antibacterial drugs is conducive to the selection of pathogens unaffected by continuous antibacterial therapy, such as *Clostridium difficile* and *C. albicans*, which may increase the susceptibility of secondary infection of fungal pathogens, increasing the morbidity and drug resistance. Therefore, there is an urgent need to exploit new treatment strategies and novel antifungal agents to manage IFI (Antinori et al., 2020).

The mammalian gut harbors a large number of microorganisms, including bacteria, fungi, archaea, protozoa, and viruses, collectively known as the microbiome. The intestinal microbial community is a dense and diverse ecosystem, whose main functions include metabolizing indigestible carbon sources, providing nutrients for the host, regulating the immune system, and preventing the invasion and colonization of pathogenic microorganisms. Thus, the destruction of the intestinal ecosystem facilitates the invasion of pathogens. For example, antibiotic treatment can kill pathogenic bacteria and cure the infection, but also inhibit the growth of other beneficial or colonizing bacteria of the same type, making it vulnerable to new pathogenic microorganisms (Kumamoto, 2016). Researches have shown that germ-free (GF) mice are more susceptible to infection than conventionally reared mice, and become less susceptible when fecal microbiota from conventional mice are transplanted with GF mice (Hooper et al., 2012).

Invasive fungi have a symbiotic relationship with bacteria in the host body to form a dynamic balance of micro-ecology, which plays an important role in the maintenance of human health (Limon et al., 2017). In the microecology, there is a mutual competition between fungi and bacteria (Zuo et al., 2018). In recent years, researches on some probiotics and intestinal commensal bacteria have shown that they can affect the colonization of invasive fungi in the intestinal tract. For example, intestinal commensal bacteria, *Enterococcus faecalis* and *Staphylococcus*, can inhibit the growth and biofilm formation of *C. albicans*. Biofilm is an important virulence factor of fungi, which endows fungi with drug resistance (Peters et al., 2012). In conclusion, the researches on probiotics and intestinal bacteria can provide new insights for clinical antifungal treatment. This review describes the antifungal activity of probiotics and intestinal commensal bacteria against the invasion and colonization of clinical common fungi, providing new strategies for the treatment of fungal infections.

## 2 The antifungal effect of probiotics

In the human body, the total amount of intestinal microorganisms are equivalent to the number of cells. Probiotics refer to “live microorganisms confer a health benefit on the host when administered in sufficient quantities” (Hill et al., 2014). The interaction between probiotics and intestinal microorganisms enables probiotics to alleviate the occurrence and development of some diseases. For example, some clinical randomized controlled trials (RCTs) have proved that probiotics can significantly alleviate the symptoms and signs related to COVID-19 such as diarrhea, headache, fever, cough, dyspnea, fatigue, myalgia and anosmia, reduce the viral load of nasopharynx, the duration of pulmonary infiltrates and the symptoms of digestive system and non-digestive system, and reduce the risk of respiratory failure and mortality (Gutiérrez-Castrellón et al., 2022). Probiotics are present not only in the intestine but also in other parts of body, such as mouth, respiratory tract, skin and genital tract. In recent years, more and more researches have shown that probiotics can exert their probiotic effects in intestinal health, immune development, nutrition metabolism, emotional management, liver diseases, oral diseases, gynecological diseases and skin health. What we need to pay special attention to is that some probiotics can inhibit the colonization of pathogenic bacteria in the intestine, strengthen the intestinal barrier, regulate the intestinal flora, synthesize active metabolites, etc. (Taverniti et al., 2021).

Clinical researches have shown that probiotics can regulate cytokine secretion, thus affecting the non-specific and specific immunity. Castrellón et al. conducted a single-center, four-blind, randomized trial on COVID-19 patients, and found that probiotic adjuvant therapy significantly increased the production of specific IgM and IgG responses to SARS-CoV-2, reduced the level of D- dimer, and reduced the risk of venous thromboembolism (such as pulmonary embolism), thus reducing the severity and mortality of COVID-19 patients (Gutiérrez-Castrellón et al., 2022). In addition, COVID-19 can cause intestinal ecological imbalance, altered intestinal permeability, and microbial translocation. However, Wu et al. found that in patients treated with probiotic adjuvant therapy COVID-19, partial recovery of intestinal ecological imbalance is evidenced by increased microbial diversity tests, and inflammatory markers such as TNF-α, IL-1β, IL-4 and IL-12P70 are also reduced (Wu et al., 2021).

Human immunodeficiency virus (HIV) infection results in altered intestinal microbiota and increased intestinal permeability, accompanied by related microbial translocation, immune activation and inflammation (González-Hernández et al., 2012). Researches have shown that probiotics can affect the immune response of people with HIV. They can avoid bacterial overgrowth and translocation by stimulating the secretion of polymeric IgA, stimulate the production of regulatory T cells by anti-inflammatory cytokines, and weaken inflammation, thus improving the immune function of HIV patients (Trois et al., 2008). In another trial, levels of D- dimer and inflammatory markers IL-6 and CRP decreased after 8 weeks of probiotic intervention with in HIV-infected subjects receiving stable antiretroviral therapy (ART), suggesting that probiotics play a beneficial role by weakening inflammation (Stiksrud et al., 2015). In addition, *Candida* colonization has been reported in more than 50% of HIV patients and symptomatic candidiasis has been reported in approximately 10% of patients. Researches have shown that the use of probiotics can reduce the number of pathogenic microorganisms (*C. albicans*, *E. coli*, *Staphylococcus aureus*, *Staphylococcus epidermidis*, and *Clostridium perfringens*) in HIV patients and tend to restore a normal range of microbial landscape (Wilson et al., 2013).

At present, the generally recognized probiotics mainly include *Lactobacillus*, Non-pathogenic *Escherichia coli*, *Bacillus*, *Enterococcus* and yeast (such as *Saccharomyces boulardii*), most of which are derived from the intestinal tract or fermented food (Zhang et al., 2022). In recent years, due to various hot researches on the intestinal microorganisms, probiotics have been proposed to antagonize opportunistic fungi and regulate the intestinal flora. On the one hand, probiotics can inhibit the invasion and colonization of pathogenic fungi by targeting their virulence factors. The researches have also revealed that the quorum sensing system is related to fungal virulence, and can be used as the target of anti-virulence treatment. On the other hand, probiotics can accumulate in the intestine and produce a variety of active metabolites (such as lactic acid, short-chain fatty acids, tryptophan, tryptophan metabolite, hydrogen peroxide, and bacteriocin, etc.) that inhibit fungal colonization (Mathur et al., 2020).

### 2.1 Virulence factor is a new antifungal target

Fungi have different pathogenic mechanisms. For example, the first step of *C. albicans* to cause infection is adhesion (Jin et al., 2005). After being swallowed by the phagocytes, yeast cells produce germ tubes and grow hypha, penetrating and destroying the host cell membrane. *C. albicans* can also produce various enzymes such as hydrolase and protease to invade and destroy tissues (Watamoto et al., 2009). *A. fumigatus* can produce some mycotoxins that damage local tissues (Rementeria et al., 2005). *C. neoformans* produces substances such as capsule, melanin, and mannitol to help the bacteria invade the host (Alspaugh, 2015). Additionally, fungi form biofilms against antifungal drugs. Therefore, researches in recent years are devoted to inhibiting or even killing fungi by targeting their virulence factors.

**Figure 1:**
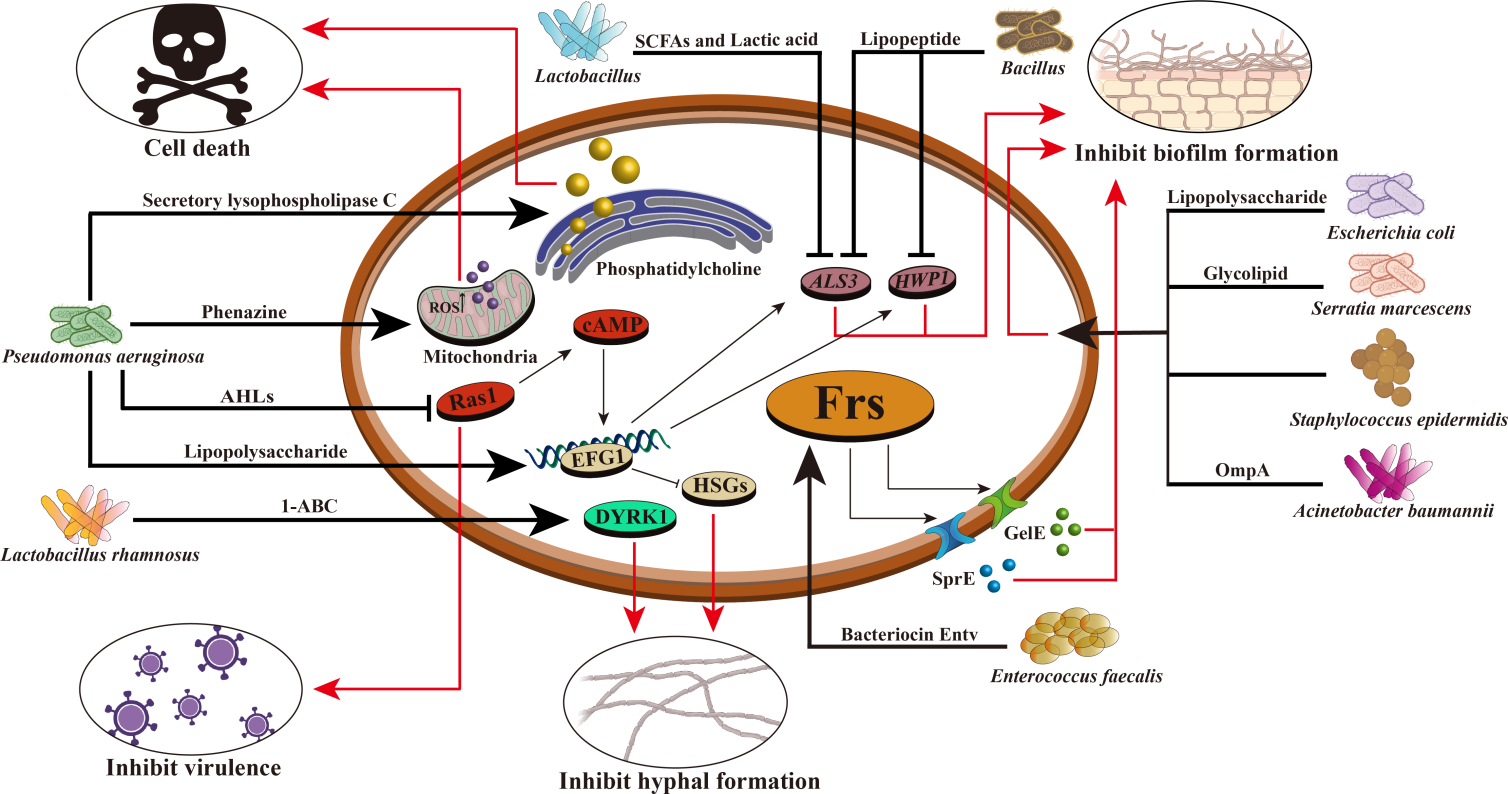
Mechanism of intestinal bacteria against *C. albicans*. Intestinal bacteria prevent the proliferation and colonization of *C. albicans* through various mechanisms. For example, *Pseudomonas aeruginosa* can produce phenazine, intracellular ROS production increased, mitochondrial membrane hyperpolarization, leading to the death of *C. albicans*; It can secrete lipopolysaccharide and improve EFG1 transcription in the hypoxia condition of biofilm environment, thereby stimulating glycolysis and inhibiting *Candida* hyphae-specific genes (HSGs), changing gene expression in biofilm formation and inhibiting hypha formation; Producing secretory lysophospholipase C, which degrades phosphatidylcholine and leads to the death of *C. albicans*; AHLs are produced that inhibit the *C. albicans* Ras1–cAMP–PKA pathway and reduce its virulence. *Enterococcus faecalis* can produce bacteriocin Entv, which can regulate the Frs system of *C. albicans*, secrete GelE, SprE and some extracellular proteases, inhibit the formation of *C. albicans* biofilm and hyphae, and reduce its toxicity. *Acinetobacter baumannii* can secrete OmpA and make it adhere to *C. albicans* to inhibit the formation of *C. albicans* hyphae and biofilm. The glycolipid substances secreted by *Serratia marcescens* have the characteristics of biosurfactant and can inhibit the formation of *C. albicans* biofilm. *Bacillus* can produce lipopeptide and also has the characteristics of biosurfactant, which can reduce the expression of *HWP1* and *ALS3* of *C. albicans* and inhibit its biofilm formation. *Lactic acid bacteria* can produce short-chain fatty acids and lactic acid, reduce the expression of *HWP1*, and inhibit the hyphal formation of *C. albicans*. *Lactobacillus rhamnosus* can secrete 1- acetyl-β-carboline (1-ABC) to inhibit the formation of *C. albicans* biofilm by inhibiting the fungal DYRK1 family kinase. *Staphylococcus epidermidis* can inhibit the biofilm formation of *C. albicans*, but its inhibition mechanism is still unclear.

Researches have shown that two new food-derived yeasts, *Saccharomyces cerevisiae* and *Issatchenkia occidentalis*, can inhibit the virulence characteristics of *candida* adhesion, filamentation and biofilm formation, indicating that food-derived yeasts can be used as an alternative antifungal therapy (Kunyeit et al., 2019). In addition, the cell-free supernatant of *L. rhamnosus* was found to reduce the virulence of *C. albicans* and inhibit the formation of its germination tubes. *L. rhamnosus* can also secrete 1- acetyl-β-carboline (1-ABC), which inhibits the transformation of *C. albicans* into mycelium and biofilm formation by inhibiting the DYRK1 family kinase of fungi, thus inhibiting its pathogenicity (**Figure 1**) (Leão et al., 2015). The antifungal mixture produced by *Lactobacillus crispatus* inhibits the growth and formation of *C. albicans* hyphae (Wang et al., 2017a). *Lactobacillus acidophilus* inhibits *C. albicans* biofilm formation and alleviates host candidiasis symptoms (Vilela et al., 2015). That is, *Lactobacillus* has inhibitory effects on fungal growth, biofilm formation, mycelium development, dental canal germination and virulence. Therefore, *Lactobacillus* can be used as a probiotic as a therapeutic adjuvant for mycosis to improve antifungal effects.

*Bacillus* is a common Gram-positive bacterium that is resistant to external harmful factors and widely distributed in soil, water, air, and animal intestinal tracts. In some ways, it can act as a probiotic to maintain the balance of microorganisms in the human body. In the human intestinal tract, co-parasitism between *Bacillus* and *C. albicans* can inhibit the invasion of *C. albicans*. Rautela’s research shows that lipopeptide secreted by *Bacillus* can inhibit the formation of fungal biofilm by reducing the expression of specific biofilm-forming genes, such as *HWP1* and *ALS3* (Rautela et al., 2014) (**Table 3**, **Figure 1**). Recent researches have shown that *Bacillus safensis* could inhibit fungal melanin exfoliation and melanosis through contact, thereby reducing *C. albicans* virulence and inhibiting biofilm formation (Mayer and Kronstad, 2017). *B. safensis* can also produce chitinase, which can damage fungal cell walls and inhibit fungal virulence factors. It is a vital active substance. Meanwhile, the virulence of other fungi (such as *C. neoformans*) could be significantly inhibited by *Bacillus* (Farrer et al., 2016) (**Table 3**). In summary, *Bacillus* reduces the fungal virulence by inhibiting the generation of hyphae, which may be a new antifungal target.

**Table 3:** Substances secreted by bacteria and their influence on fungi.

	Invasive fungi	Produced by bacteria	Mechanism of action	Functional effect	References
*Pseudomonas aeruginosa*	*Candida albicans*	Phenazine	Oxidative activity, intracellular ROS production increased, mitochondrial membrane hyperpolarized, affect *C. albicans* respiration, apoptosis.	Inhibit metabolism and biofilm formation	(Gibson et al., 2009; Tupe et al., 2015)
		Lipopolysaccharide	LPS up-regulated the expression of transcription factor EFG1 in *C.albicans* biofilm, increased glycolysis and inhibited *Candida* hyphae-specific genes (HSGs)	Alter gene expression during biofilm formation and inhibit hyphal formation	(Bandara et al., 2013)
		Secretory lysophospholipase C	Degraded phosphatidylcholine (phospholipids abundant in eukaryotes)	The death of the fungal cell	(Hogan and Kolter, 2002)
		Acyl homoserine lactones (AHLs)	Inhibit the Ras1–cAMP–PKA pathway for hyphal growth in *C. albicans*	Inhibit virulence	(Davis-Hanna et al., 2008)
	*Cryptococcus neoformans*	Pyocyanine、2-heptyl-3,4-dihydroxyquinoline (PQS)	*Pseudomonas aeruginosa* contacts *C.neoformans*, and antifungal molecules play a role	Inhibit growth	(Rella et al., 2012)
	*Aspergillus fumigatus*	Pyocyanine	Chelated iron, deprived of *A.fumigatus* necessary nutrition for growth and metabolism	Inhibit virulence	(Mowat et al., 2010)
*Staphylococcus*	*Candida albicans*	–	–	Inhibit biofilm formation	(Adam et al., 2002)
	*Cryptococcus neoformans*	–	*Staphylococcus aureus* attached to *cryptococcus capsulatus*, mitochondrial pathway was enhanced, and cells died.	Apoptotic cell death	(Ikeda et al., 2007)
	*Aspergillus fumigatus*	–	Fungal growth may be significantly limited by intercellular contact and synthesis of bacterial products	Inhibit growth, conidia, hypha and biofilm formation	(Ramírez-Granillo et al., 2021)
*Bacillus*	*Candida albicans*	Lipopeptide	Biosurfactant properties, reduced the mRNA expression of hypha-specific genes *HWP1* and *ALS3*	Inhibit biofilm formation	(Ceresa et al., 2016; Janek et al., 2020)
	*Cryptococcus neoformans*	Chitinase Laccase	Reduce the structural stability of cell wall to inhibit capsule formation; Inhibit melanin formation	Inhibit biofilm formation and virulence factor production	(Farrer et al., 2018; Upadhya et al., 2018)
	*Aspergillus fumigatus*	–	Inhibit the gene expression related to hyphae	Inhibit hyphae formation	(Benoit et al., 2015)
*Enterococcus faecalis*	*Candida albicans*	Bacteriocin Entv	Regulate that Frs system of *Candida*, secreting GelE, SprE and some extracellular protease	Inhibit the formation of hyphae and biofilm, inhibit virulence	(Cruz et al., 2013)
*Lactobacillus*	*Candida albicans*	Short-chain fatty acid Lactic acid	Inhibit the expression of *C. albicans HWP1* gene, reduce the adhesion and the stability of biofilm structure	Inhibit virulence and hyphae formation	(Noverr and Huffnagle, 2004; Jang et al., 2019)
	*Cryptococcus neoformans*	Sodium butyrate	Inhibit the formation of capsule and melanin	Inhibit growth and enhance macrophages	(Hoberg et al., 1983)
*Escherichia coli*	*Candida albicans*	Lipopolysaccharide	–	Modulate biofilm formation	(Bandara et al., 2010)
	*Aspergillus fumigatus*	Cytosolic proteins	Inhibit the development of conidia and cause hyphal atrophy	Inhibit growth	(Balhara et al., 2014)
*Acinetobacter baumannii*	*Candida albicans*	Outermembrane protein A(OmpA)	OmpA Attaches *A. baumannii* to *C.albicans* Filament	Inhibit hyphae and biofilm formation	(Brossard and Campagnari, 2012)
*Serratia marcescens*	*Candida albicans*	Glycolipid	Biosurfactant properties, anti-adhesion	Inhibit biofilm formation	(Dusane et al., 2011)

“-” No detailed reports at the time of review

A key regulatory hub for virulence is quorum sensing (QS). QS is a signaling transmission mechanism between microorganisms, through the secretion and release of some specific signal molecules, the perception of their concentration changes to monitor the population density, regulate the physiological function of the population, so as to adapt to the surrounding environment. It is also called “intercellular communication” or “self-induction”. The signaling molecules produced during the regulation are also called autoinducers (AI) (Galloway et al., 2011). Researchers found that QS regulates biological behaviors of bacteria, such as fluorescence production, toxin production, and the production of extracellular enzymes of pathogenic bacteria, especially biofilm formation (Miller and Bassler, 2001; Hooshangi and Bentley, 2008; Yang and Defoirdt, 2015). For example, *S. aureus* requires the Agr quorum sensing system to achieve intestinal colonization, and the lipopeptide fencarinin produced by *Bacillus* (mainly *Bacillus subtilis*) has a similar structure to AIP (auto-inducing peptide), which is a key factor in Agr quorum sensing and can interfere with Agr signal transduction, thereby inhibiting Agr quorum sensing and ultimately preventing *S. aureus* from colonizing the intestinal tract to eliminate this notorious multi-antibiotic-resistant bacterium (Piewngam et al., 2018). Moreover, quorum sensing systems exist not only in bacteria but also in fungi.

Farnesol in *C. albicans* is the first discovered quorum sensing molecule (QSM) (Hornby et al., 2001). Researches have shown that farnesol can inhibit *C. albicans* biofilm formation, can inhibit the transformation of *C. albicans* from yeast type to mycelium type, and can also induce the expression of antioxidant genes to resist oxidative stress (Langford et al., 2009). The Ras1 cyclic AMP protein kinase A pathway positively regulates *C. albicans* mycelial growth, and farnesol inhibits mycelial growth by inhibiting elements in this pathway (Davis-Hanna et al., 2008). 2-phenylethanol (2-PE) is a QSM in *S. cerevisiae*. Researches have shown that exogenous 2-PE could stimulate biofilm formation at low cell concentrations. In addition, 2-PE is synthesized mainly by the Ehrlich pathway, involving *ARO8p* and *ARO9p*, which are essential for the formation of biofilm. This research screened the mutants *ΔARO8* and *ΔARO9*, and found that the deletions of these two genes reduced the 2-PE content, ethanol yield, extracellular polysaccharide content, the expression of *FLOp* participating in cell adhesion in the early stage of fermentation, and decreased the formation of biofilm (Wang et al., 2017b; Zhang et al., 2021). These findings suggest that the production of 2-PE could promote the formation of *S. cerevisiae* biofilm, so and thus the formation of biofilm could be inhibited by reducing the production of 2-PE. Other fungi also have quorum sensing signal molecules, such as *C. neoformans*, with an 11-amino acid polypeptide signaling molecule that promotes fungal growth on solid media (De Sordi and Mühlschlegel, 2009). All these indicate that the quorum sensing system plays an important role in fungi, but the signaling molecules and mechanisms of most fungi are still unclear. However, in the future antifungal therapy, we can study the mechanism of QS in fungi to understand the pathogenic mechanism, and use the quorum sensing system as a target for fungal anti-virulence therapy to develop new antifungal drugs.

### 2.2 Probiotics produce active metabolites to inhibit fungi

Among a variety of active metabolites produced by probiotics, bacteriocin is a 20-60 amino acid long polypeptide or protein with antibacterial activity synthesized by bacteria through the ribosomal pathway during the metabolic process. It is safe, nontoxic and has strong antibacterial activity, and is an important active metabolite (Donia and Fischbach, 2015). At present, it is generally believed that bacteriocin can destroy the cell membrane structure, form pores in the cell membrane, leak out intracellular substances, inhibit the function of the proton pump, cause the disorder of membrane potential and pH gradient, and then lead to the inhibition of DNA, RNA, protein and polysaccharide synthesis. It causes the loss of intracellular nutrients and eventually leads to bacterial cell death, thereby inhibiting the growth of intestinal pathogens and regulating the balance of intestinal flora (Moll et al., 1999). For example, *E. faecalis* can secrete a bacteriocin, Entv, which can inhibit the virulence, hyphal and biofilm formation of *C. albicans*, and thus inhibit its colonization and invasion of the intestinal tract (**Table 3**, **Figure 1**) (Graham et al., 2017). *L. rhamnosus* L60 and *Lactobacillus fermentum* L23 have potential probiotic activities, and their secondary active metabolite, bacteriocin, can inhibit the biosynthesis of aflatoxin B1 (AFB1) and the growth of *A. flavus* (Gerbaldo et al., 2012). Moreover, bacteriocin has a unique antibacterial mechanism, which is not easy to cause drug resistance; It has low immunogenicity and it is not easy to generate immune reaction in human body; It can be decomposed by protease in human body, so it is less toxic to the human body and will not destroy normal intestinal ecology (Barbosa et al., 2015). Therefore, probiotics that secrete bacteriocins that have specific antibacterial effects without disrupting the normal intestinal flora may be more desirable antibacterial agents than antibiotics.

According to related reports, lactic acid is considered as an important antibacterial substance that can lower the pH of fermentation broth, creating an acidic environment, which is not conducive to the growth of pathogenic bacteria. In addition, lactic acid can also enter the cell membrane and cannot be excreted, acidifying the cytoplasm of bacteria, interfering with intracellular functions and leading to cell death, thereby achieving a strong antibacterial effect (Tachedjian et al., 2017). Vulvovaginal candidiasis (VCC) is one of the major causes of female genital infections, and its main pathogenic factor is *C. albicans*. Clinically, fluconazole is the first choice for the treatment of VCC, but the increasing resistance of *Candida* strains to this treatment has prompted the search for alternative therapies for VCC (Rodríguez-Cerdeira et al., 2019). It is well known that lactic acid bacteria can ferment sugar to produce large amounts of lactic acid and obtain energy. For example, researches have confirmed that *Lactobacillus casei* can inhibit the growth of *C. albicans* causing VCC, and reduce the formation of *C. albicans* biofilm and hyphae, reducing their drug resistance and preventing invasive systemic infection (Paniágua et al., 2021). DRutz et al. also found a protective effect of oral administration of *L. acidophilus* on candidal vaginitis (Drutz, 1992). There are also researches that show that after women are infected with HIV, lactic acid produced by lactobacillus can acidify the vagina and reduce the burden of HIV, thus reducing the risk of HIV transmission among women like men and reducing the colonization of some opportunistic pathogenic *Candida* (Tachedjian et al., 2017).

Besides, short-chain fatty acids (SCFAs: such as acetate, propionate and butyrate) produced by probiotics can change the pH environment of the intestine. For example, butyrate can reduce the expression of several virulent genes of *Salmonella enterica* and *Salmonella typhimurium* and inhibit the growth of enterohemorrhagic *Escherichia coli* (EHEC) (Shin et al., 2002). SCFAs secreted by *Lactobacillus* can inhibit the morphological transformation of *C. albicans* and reduce its pathogenicity (Liang et al., 2016). They can affect the formation of hyphae and pseudohyphae by affecting the expression level of the *HWP1* gene of *C. albicans*, reduce adhesion activity, and lose structural stability of biofilm (Matsuda et al., 2018; Rossoni et al., 2018). Furthermore, *Lactobacillus* can produce butyrate, which can inhibit filamentation of *C. albicans*, block the formation of capsule and melanin of *C. neoformans*, and enhance the effector function of macrophages (Nguyen et al., 2011). In addition, SCFAs can be used as hosts to reduce oxygen concentration and create an environment that is not conducive to fungal growth (Rivera-Chávez et al., 2016). Probiotics can also inhibit pathogen adhesion to intestinal epithelium by producing bacteriocins and SCFAs; improve the barrier function of intestinal mucosa by increasing mucus layer and producing tight junction proteins; stimulate the production of secretory IgA to improve intestinal immunity, and then relieve metabolic diseases and intestinal disorders diseases such as IBD, IBS and colorectal cancers (Sendid et al., 2009; Eslami et al., 2019; Simon et al., 2021).

Indole, a metabolite of tryptophan, is usually produced by intestinal bacteria containing tryptophanase, and is a specific intestinal commensal bacterial signal (Beaumont et al., 2018). Indole is important for innate immunity, inhibition of inflammation, elimination of free radicals and maintaining the integrity of intestinal immune barrier (Roager and Licht, 2018). Researches have shown that commensal *E. coli* can produce indole to inhibit the chemotaxis of pathogenic *E. coli*, and inhibit the adhesion of pathogenic bacteria to the intestinal epithelium by increasing the expression of genes involved in intestinal epithelial function, such as actin cytoskeleton and adhesive junction (Bansal et al., 2007). In addition, indole can be used as a ligand for aryl hydrocarbon receptor (AhR) to mediate intestinal immune regulation, which is beneficial to immune homeostasis and affects host physiological function. *Bifidobacterium infantis* has been reported to produce indole -3- lactic acid, which activates the AhRs of the intestinal epithelium by increasing its upregulation of CYP1A1 protein expression. The activation of AhR leads to the transcription of lL-22. In the intestinal tract, lL-22 can regulate the release of antimicrobial peptides, further increase the expression of antimicrobial peptides, and reduce the colonization of *C. albicans* in the gastrointestinal tract (Liu et al., 2020). Another research also showed that the cell-free supernatant of *Lactobacillus paracasei* contained metabolites such as lactic acid, SCFAs, indoleacetic acid and 2- hydroxy -3- methylbutyric acid, etc., which had good antifungal activity (Honoré et al., 2016). After these metabolites secreted by probiotics, including probiotics, regulate the intestinal flora, the changes of microbial metabolites in the intestinal tract will eventually affect the intestinal barrier, and the permeability of the intestinal barrier will be reduced, and our general inflammation will also be reduced, thus greatly reducing our infection rate and prevalence rate. These active metabolites are considered to be a molecule derived from natural resources, which are less susceptible to drug resistance than the high cost and unpredictability of developing new antifungal agents, is a feasible and promising approach.

Whether the probiotics themselves are used to inhibit the virulence factors of fungi, or anti-virulence treatment by targeting the quorum sensing system of fungi, or the active metabolites secreted by probiotics to inhibit the invasion and colonization of fungi in the intestine, all these have shown that probiotics can promote human health. Therefore, the potential of probiotics inspires more people to explore the untapped microorganisms in the healthy human gut to fight fungi.

## 3 Using intestinal commensal bacteria to inhibit the invasion of pathogenic fungi

At present, it has been confirmed that in addition to some recognized probiotics, some intestinal commensal bacteria can also inhibit the invasion of fungi through a certain mechanism, including Gram-positive and Gram-negative bacteria, such as *Staphylococcus*, *Pseudomonas aeruginosa* and *E. coli*, etc.

*Staphylococcus* is a common Gram-positive coccus that can exist on the human body surface, mouth and throat and inside the intestine, mainly causing nosocomial cross-infection. Human infection with *Staphylococcus* can cause severe diseases such as pneumonia, sepsis, and toxic shock syndrome, and even lead to death. But researches have found that *Staphylococcus* can inhibit the growth of *C. albicans*, when they are dominant in the niche. Culture supernatant of *S. epidermidis* has been proved to inhibit *C. albicans* biofilm formation, but the mechanism of inhibition is still unclear (Bhattacharyya et al., 2014). When *S. aureus* and *C. neoformans* are co-cultured, they can contact with each other to inhibit the growth of *C. neoformans*. When the two organisms are not in contact, inhibition of *C. neoformans* disappears (Saito and Ikeda, 2005). The mechanism is that *S. aureus* enhances the mitochondrial pathway of *C. neoformans* by attaching to the capsule of *C. neoformans*, resulting in the death of *C. neoformans* (Ikeda, 2011). *S. aureus* has been found to significantly inhibit the formation and development of *A. fumigatus* biofilm *in vitro*, regardless of the amount of bacterial inoculum with *A. fumigatus* (Ramírez Granillo et al., 2015). In general, *C. albicans* and *C. neoformans* can be inhibited by *S. aureus* when they are in contact with *S. aureus* (Saito and Ikeda, 2005).

In addition to Gram-positive bacteria, some Gram-negative bacteria can also inhibit the virulence factors of invasive fungus or directly inhibit their growth. For example, *P. aeruginosa*, a Gram-negative bacterium found in various water, air, normal human skin, respiratory tract, and intestinal tract, and is also an opportunistic pathogen. Human infection can cause postoperative wound infection, abscess, bacteremia and other symptoms, and even lead to death. Clinically, mixed infections of *P. aeruginosa* with *C. albicans*, *A. fumigatus*, and *C. neoformans* have been found in patients with pulmonary fibrosis (Nazik et al., 2017). But some experiments show that *P. aeruginosa* can inhibit *C. albicans* infection. *P. aeruginosa* can produce phenazine to inhibit the metabolic process and *C. albicans* biofilm formation (Morales et al., 2013). Therefore, the combination of azoles and phenazine can enhance the efficacy of candidiasis and provide a new idea for clinical treatment of *Candida* infections (Nishanth Kumar et al., 2014). In addition, *P. aeruginosa* can secrete lipopolysaccharide, which can inhibit the formation of *C. albicans* hyphae and biofilm formation by affecting the expression of key genes during biofilm formation (Bandara et al., 2013). It is recognized that Fe^2+^ and Cu^2+^ are indispensable nutrients for the growth of *C. albicans*. However, *P. aeruginosa* can inhibit fungal growth by inhibiting the fungal absorption processes of Fe^2+^ and Cu^2+^ (Robinett et al., 2019). Similarly, *P. aeruginosa* produces pyocyanin that inhibits the growth of *C. neoformans* when contacts with *C. neoformans* (Rella et al., 2012). The pyocyanin can also inhibit *A. fumigatus* growth, resulting in a deficiency of metal iron ions in the bacterium (Sass et al., 2018). Based on the current researches, we found that pyoverdine is the key substance for inhibiting the growth of fungi, and its inhibitory effect is regulated by the metabolism of metal ions, such as Fe^2+^ and Cu^2+^ (Hunsaker and Franz, 2019).

Besides *P. aeruginosa*, there are some Gram-negative bacteria that can inhibit the growth of invasive fungi. For example, *E. coli* can secrete lipopolysaccharide to inhibit the formation of *Candida* biofilm (Bandara et al., 2009). In addition, a specific protein produced by a variety of *Escherichia* bacteria can inhibit the development of conidia and cause hyphal atrophy, which in turn inhibits the growth of *A. fumigatus* (Yadav et al., 2005). *Acinetobacter baumannii* shows antifungal activity by binding to *C. albicans* hyphae and inducing apoptosis to prevent its biofilm formation through outer membrane protein (OmpA) (Gaddy et al., 2009).

Some intestinal commensal bacteria can also defend fungi by regulating the host anti-fungal immune response. For example, Villena et al. in a mouse model infected with *Candida* have found that supplementation of *L. casei* diet to mice can reduce mortality, increase phagocytosis and fungicidal activity of intestinal macrophages, and increase the number and function of neutrophils to prevent colonization and invasion of *Candida* (Villena et al., 2011). Researches have confirmed that *Bacteroides fragilis* produces the molecular polysaccharide A(PSA), which can eliminate fungi and maintain the steady state of the intestinal environment by inducing anti-inflammatory regulatory T cells (Treg cells) through TLR2 stimulation (Round et al., 2011). To sum up, intestinal commensal bacteria are essential to maintain the intestinal microecological homeostasis, and at the same time, some intestinal commensal bacteria can be used to prevent and control IFI. Although this approach still has great limitations, it is a vital step in developing new antifungal strategies.

## 4 Conclusion and prospect

In recent years, IFI have increased, together with the increase in drug resistance and the limited variety of antifungal drugs, which have posed an unprecedented threat to public health. Therefore, we need new strategies to fight IFI. There is a great deal of microbial symbiosis in our gut, including bacteria, fungi, viruses, archaea, bacteriophage and protozoa. Microbes settle down immediately after the birth of mammals, and many microbes that reside in the intestinal tract adapt to the intestinal environment and compete with other microbes for the nutritional niche of the host. Moreover, more and more researches have focused on the use of intestinal bacteria against fungi, and they have been confirmed that some probiotics and intestinal commensal bacteria can inhibit fungal growth, invasion, and gut colonization.

Besides some of the traditional probiotics mentioned above, “next-generation probiotics” are beginning to emerge as new preventive and therapeutic tools, such as *Faecalibacterium prausnitzii* and *Akkermansia muciniphila* (O’Toole et al., 2017). Researches have shown that oral administration of live *F. prausnitzii* or its supernatant significantly reduces the severity of TNBS colitis, in part because it secretes metabolites that prevent NF-κB activation and IL-8 production (Sokol et al., 2008). Although there are few researches on the next generation of probiotics against pathogenic fungi, but Faecalibacterium can produce high levels of butyrate, and whether it inhibits fungi like other intestinal probiotics needs to be explored.

However, the use of intestinal bacteria against IFI also has certain limitations. For example, the mechanism by which some intestinal symbionts affect fungal colonization is still unclear and needs to be further studied, which is also a promising direction for future researches on intestinal bacteria. Whether or how they can be applied clinically for antifungal therapy is also a problem worthy of deep thinking. In addition, the protection provided by microorganisms is highly dependent on the host’s diet, and probiotic-derived metabolites need to be consumed specific nutrients before they are produced. For example, SCFAs-butyrate and propionate are derived from the fermentation of fibers in the colon. Classical western-style diet usually lacks fruits and vegetables (sources of fiber and flavonoids), resulting in decreased abundance of intestinal bacteria and reorganization of intestinal microflora (Wastyk et al., 2023). At the same time, the intestinal flora deprived of dietary fiber damages the colonic mucus barrier, thus increasing pathogen susceptibility (Desai et al., 2016). All in all, the intestinal flora and the human body are mutually beneficial and inseparable. It is an important place for probiotics and some intestinal commensal bacteria to exert an antifungal effect. In combination with the rapidly developing omics methods, we can explore the antifungal probiotics and commensal bacteria in the intestine. In-depth study of their antifungal mechanism will provide new ideas and treatment strategies for “fungal inhibition by bacteria” in the future against pathogenic fungi.

## Author contributions

Conceptualization, YL and ZZ; methodology, YL, LC, and ZZ; software, LC and SM; validation, LC, CC, and ZH; investigation, SM; writing—original draft preparation, LC; writing—review and editing, LC, CC, and ZH; supervision, YL and ZZ; funding acquisition, YL and ZZ. All authors contributed to the article and approved the submitted version.
